# The underlying molecular mechanism of ciliated epithelium dysfunction and TGF-β signaling in children with congenital pulmonary airway malformations

**DOI:** 10.1038/s41598-024-54924-x

**Published:** 2024-02-23

**Authors:** Gang Zhang, Lei Lou, Linghui Shen, Huiyi Zeng, Chun Cai, Rongde Wu, Dandan Liu

**Affiliations:** 1 Department of Pediatric Surgery, Shandong Provincial Hospital, Shandong University, Jinan, 250021 Shandong China; 2 Medical Integration and Practice Center, Shandong University, Jinan, 250012 Shandong China; 3 Department of Pediatric Surgery, The Third Affiliated Hospital of Guangzhou Medical University, Guangzhou, 510150 Guangdong China; 4 Department of Pediatric Surgery, Shandong Provincial Hospital Affiliated to Shandong First Medical University, Jinan, 250021 Shandong China; 5 Department of Fetal Medicine and Prenatal Diagnosis, The Third Affiliated Hospital of Guangzhou Medical University, No. 63, Duobao Road, Liwan District, Guangzhou, 510150 Guangdong China

**Keywords:** Congenital pulmonary malformation, RNA-Seq, SMAD6, TGF-β, Respiration, Molecular biology, Transcriptomics

## Abstract

The aim of this study was to investigate the variation in gene expression in the complete transcripts of Congenitalpulmonary airwaymalformation (CPAM) of the lung using Next Generation Sequencing (NGS) technology. There were 20 cases involving children with CPAM were used for selection of study sample. NGS was used to establish RNA-Seq libraries for the two groups of samples separately, and both groups were conducted to differential expression analysis and Gene Ontology (GO) functional enrichment analysis. The pathways of the differential genes were analyzed to find the enriched target pathways. A total of 592 genes were expressed with significant differences (CPAM vs. normal tissue, *P* < 0.05). GO functional analysis of DEGs indicated that abnormal ciliary function played a role in the development of CPAM. Subsequently, analysis of these genes pathways showed the TGF-β signaling pathway was significantly enriched. Finally, the results of immunohistochemical analysis of some DEGs showed that a significant reduction in the expression of SMAD6, a gene related to the TGF-β signaling pathway, led to abnormal activation of the pathway. TGF-β signaling pathway involved in the evolution of the disease obtained by DEGs enrichment pathway analysis. SMAD6, a gene involved in this pathway, might be a potential biomarker for the diagnosis and treatment of CPAM.

## Introduction

Congenital pulmonary malformation (CPAM) is a rare lung malformation, and accounts for approximately 30–40% of congenital lung disease and 1/25,000 to 1/35,000 in live births^1,2^. It is characterized by an unusual airway patterning and different degrees of pseudo-cystic dilatation of airways with unclear nosogenesis^3^. It leads to defective development of fetal lungs with mediastinal shift, neonatal respiratory distress, pulmonary edema, lung infections, and even the development of malignant tumors^4^. However, as prenatal diagnostic techniques continue to progress, the rate of their detection has been increasing^5^. Most patients with CPAM are detected at prenatal diagnosis or in the neonatal period^6^. Currently, routine prenatal US screening complemented with magnetic resonance imaging (MRI) has become increasingly valuable in detecting CPAMs, with an accuracy of 65–91%^7^.

With the rapid development of genomics and bioinformatics, a large amount of genomic data is being analyzed. Due to the complex pathophysiology and the heterogeneity of cells in lung tissues, it is necessary to have a comprehensive insight into the mRNA expression of CPAM. Many researchers have devoted themselves to the study of CPAM, several factors such as fibroblast growth factor-10 (FGF-10)^8^, FGF-2^9^, transforming growth factor-β (TGF-β)^10^ and other factors associated with lung development have been identified. However, the genetic landscape of CPAM is poorly known compared to other congenital lesions, and its introduction into biologically-driven risk stratification approaches and new drug development has been relatively slow.

This study aimed to analyze the genomic characteristics and the target signaling pathway involved in the development of CPAM. Potential biomarkers were also explored in the development of CPAM.

## Material and methods

The study was approved by The Third Affiliated Hospital of Guangzhou Medical University (NO. 202030). All described experiments were performed in accordance to the relevant guidelines and regulations set forth by the IRB. Written informed consents were obtained from both groups.

### Sample collection

**Figure 1:**
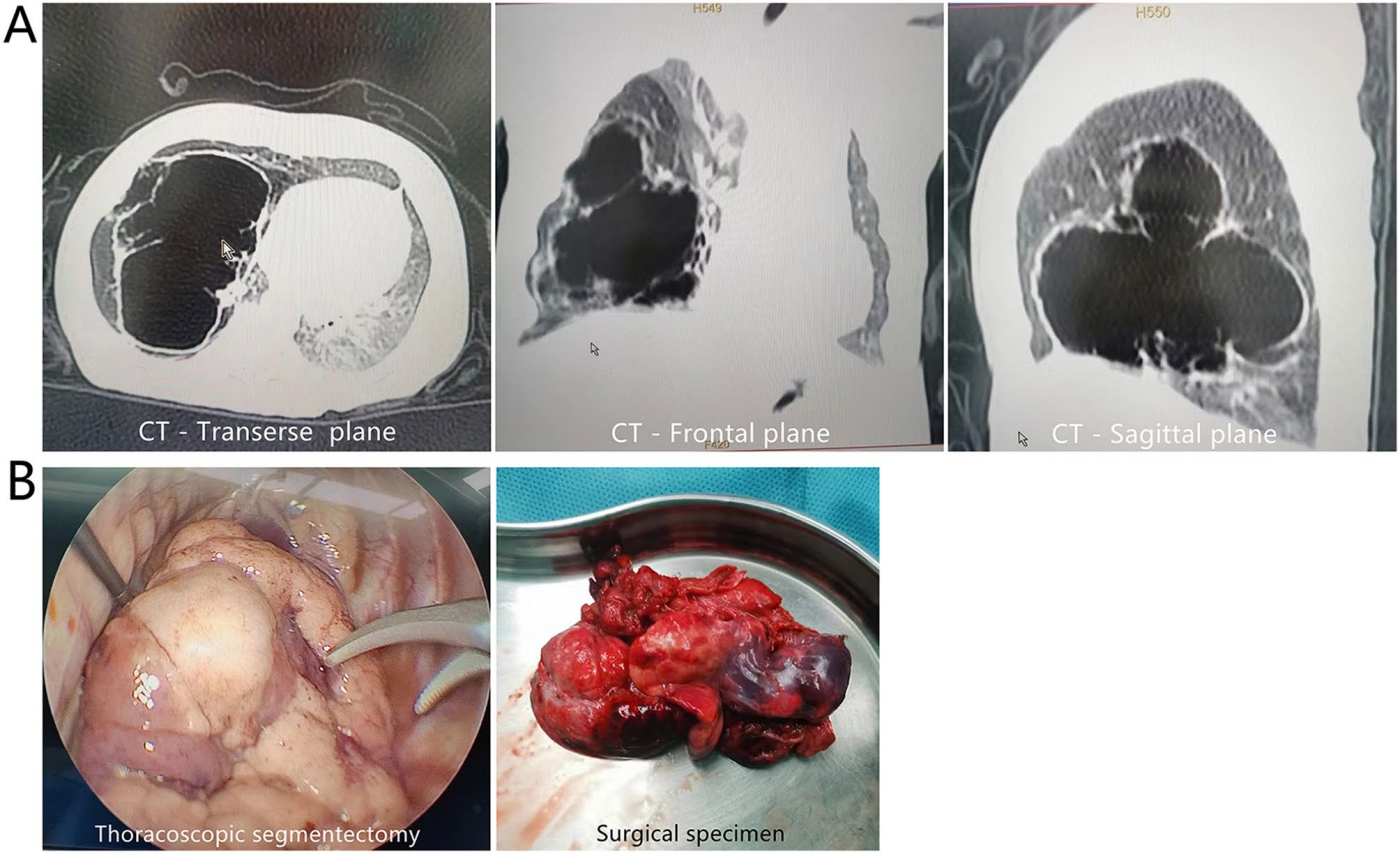
In case 4, a 3-month-old male infant were diagnosed with a suspected right-sided CPAM based on an abnormal 20-week prenatal ultrasound. A chest computed tomography (CT) scan was performed prior to surgery (**A**), revealing a massive right-sided cystic lesion measuring 63 × 52 × 49 mm, along with poor ventilation of the remaining lungs. The patient underwent a thoracoscopic procedure, wherein the dorsal and basal segments of the right lower lobe were excised (**B**).

We collected tissue samples from a total of 20 patients, including 10 cases of histopathologically confirmed CPAM and 10 cases of normal lung tissue (NL) adjacent to other lung lesions from patients who underwent surgery due to pulmonary diseases since 2019. In case 4, a 3-month-old male infant were diagnosed with a suspected right-sided CPAM based on an abnormal 20-week prenatal ultrasound. A chest computed tomography (CT) scan was performed prior to surgery (Fig. 1A), revealing a massive right-sided cystic lesion measuring 63 × 52 × 49 mm, along with poor ventilation of the remaining lungs. The patient underwent a thoracoscopic procedure, wherein the dorsal and basal segments of the right lower lobe were excised (Fig. 1B). Postoperative histopathological examination confirmed the diagnosis of CPAM.

NL samples were acquired from patients who underwent surgery for non-malignant lung conditions, with histopathological confirmation of normal lung tissue. Tissue samples were immediately preserved using two different methods: Rapid freezing in liquid nitrogen and storage at − 80 °C. Fixation in formalin followed by paraffin embedding for histological analysis.

### RNA seqencing

The 6 groups of samples collected in the early stage were offered RNA sequencing. The total RNA extraction was performed using TRIzol reagent (Invitrogen, Carlsbad, CA, USA). Subsequently, the concentration and purity of the extracted RNA were assessed using a Nanodrop ND-2000 spectrophotometer (Thermo Fisher Scientific, Wilmington, DE, USA). RNA quality standards were set as follows: a quantity greater than 5 µg and a concentration of at least 200 ng/µL. The RNA integrity number (RIN) value, exceeding 7, was determined using an Agilent 2100 Bioanalyzer (Agilent, Palo Alto, CA, USA).

To eliminate ribosomal RNA (rRNA) from the total RNA samples, the Ribo-Zero Gold rRNA removal kit (Illumina) was employed. Subsequently, the RNA was fragmented into 200 bp fragments using the total RNA SEQ (H/M/R) library prep kit (Illumina, San Diego, CA, USA). The fragmented RNA was then used for complementary DNA (cDNA) synthesis. After purification, end repair, adapter ligation, A-tail addition, product purification, and fragment size sorting, library amplification was carried out. Following amplification, the RNA SEQ library underwent purification and recovery using magnetic beads. The constructed library was subjected to inspection, and the qualified libraries were pooled based on data size and effective cDNA concentration.

The sequencing of the library was performed on the IlluminaNovaSeq 6000 platform in paired-end 150-bp mode. Raw data underwent filtration to eliminate low-quality reads and reads containing adapter or Poly-N sequences. Clean reads were then aligned to the Ribosomal Database Project using Bowtie, and reads corresponding to ribosomal DNA (rRNA) were subsequently removed. The reads mapped to the reference sequence were utilized for mapping, sequence prediction, calculating expression values, and conducting expression difference analyses.

### Differential gene expression analysis

**Figure 2:**
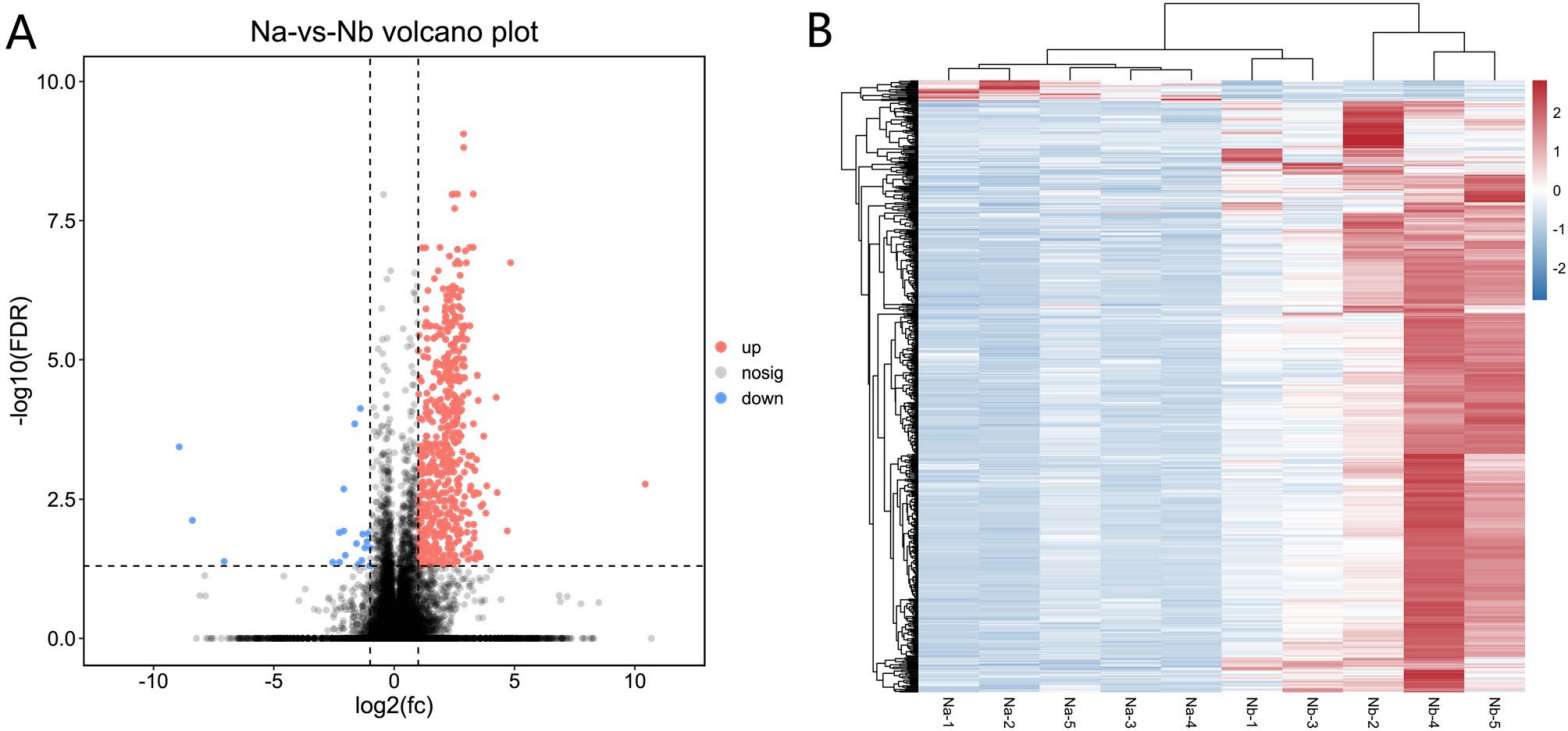
Differentially expressed genes (DEG) analysis. (**A**) The Volcano Plot illustrates the relationship between gene expression fold change and significance, highlighting significantly upregulated (red) and downregulated (blue) genes; (**B**) the Heatmap displays gene expression levels across various conditions, with red colors indicating higher expression and blue colors indicating lower expression.

With the utilization of the R package limma, we detected a total of 592 differentially expressed genes (DEGs) in CPAM. Among these, 572 genes exhibited upregulation, while 20 were observed to be downregulated (Fig. 2A, B).

### GO and KEGG pathway analysis of DEGs

GO (geneontology.org) and KEGG (genome.jp/kegg) analyses of differentially expressed lncRNA-associated genes were performed using the online Database for Annotation, Visualization and Integrated Discovery tool. The top 10 enriched GO terms among the two groups were identified. GO analysis results consisted of ‘biological process’ (BP), ‘cell composition’ (CC) and ‘molecular function’ (MF). The adjusted *P* value was also obtained using the Benjamini & Hochberg method and an adjusted *P* < 0.05 was considered to indicate a statistically significant difference. The pathways associated with lncRNA-targeted mRNAs were identified by KEGG pathway analysis^11^.

### Quantitative real-time PCR

RNA extraction from 5 CPAM samples and 5 normal lung tissues using TRIzol (Tel-Test, Friendswood, Tex). cDNA synthesis from 1 μg RNA using Superscript III (Invitrogen, Carlsbad, Calif). Real-time quantitative PCR was performed on cDNAs using the Quanti-Tect SYBR Green PCR Kit (Qiagen, Valencia, Calif) and an ABI Prism 7000 (Applied Biosystems, Foster City, Calif). The following primers were used:

SMAD6-F:AGTGACTGCGAGACGGTGACC R:GCAGCGAGTACGTGACGGTTT;

RANBP9-F: ACGAGCAGGAGAAGGAGTTGC R: TGGTATTGGATGCGTGGCTCG;

DNAH2-F: GCTGTCAGTGAGCCAGAGCTG R: CTGTGTCCACACTGCATCCGC;

DNAH11-F: CGGAATTCCCACAGACACGCA R: CAGCCACCTGCACCTTTTCCA;

CFAP43-F: TTGTGGGCGTCATGGCAACTA R: GCCAGGTAGGTGCCACAGTAA;

CFAP46-F: CCCATCACCCAGTTTCTGGGC R: CTTGAGGAACGGCCTCACCAT;

RP1-F: AAGAGCGGAGACCCCCAATTC R: TAGGTAGGACTCGCCGTCCTC;

CFAP77-F: ATCCGTTCCGGCATGGAGAAC R: GATGGCTTCAGGGACTCCTCC;

RSPH4A-F: GCCAGAAACTGGACGCCAGTC R: CTCGTGGTCCTGTCCGACTGA;

DRC3-F: CCGAGGGTGATGGACGATGAC R: CTGGGAATCCTCCTGGCCATC.

Relative abundance of RNA was calculated by the ΔΔCt method. Primers were designed using Primer Express v2.0 (Applied Biosystems, Foster City, Calif). All primers were between 90 and 110% efficient, as assessed by standard curve, and all displayed only 1 dissociation peak.

### Immunohistochemistry

The remaining portion of the sample was analyzed utilizing immunohistochemistry. Immunohistochemical staining procedures were carried out in accordance with the manufacturer's provided instructions. Primary antibodies utilized for staining were as follows: rabbit anti-SMAD6 (dilution 1:100, Abcam, Cambridge, UK), rabbit anti- InhibinB (dilution 1:100, Abcam, Cambridge, UK), and rabbit anti-GDF7 (dilution 1:2000, Abcam, Cambridge, UK).

The mean optical density of positively stained cells was quantified using ImageJ software (version 1.53e). This quantification was based on the optical density of stained positive cells, which was then normalized to the total cell area within each field of view at 200× magnification.

### Statistical analysis

Statistical analyses were conducted utilizing GraphPad Prism (version 7) and R packages. For comparisons between independent groups, the Student's *t* test was applied, while paired groups were assessed using the paired *t* test. Continuous variable results were expressed as mean ± standard deviation. A significance threshold of *P* ≤ 0.05 was used to determine statistical significance.

All the data collected in this study were analyzed using GraphPad Prism (version 7) and R software 4.0. Normally distributed measurement data were expressed as mean ± standard deviation (SD), and the comparisons were examined by Student-*t* test. *P* < 0.05 was considered statistically significant.

### Ethics approval and consent to participate

The study was approved by The Third Affiliated Hospital of Guangzhou Medical University. (NO. 202030). All described experiments were performed in accordance to the relevant guidelines and regulations set forth by the IRB. Written informed consents were obtained from all patients.

## Results

### Identification of differentially expressed genes in CPAM

All cases of CPAM with detailed clinical information were recorded in Table 1. In this study, we conducted RNA-seq analysis on CPAM tissue samples and normal lung tissues, identifying a total of 592 differentially expressed genes (|FoldChange|> 2 and q-value < 0.05. DEGs included both upregulated (572) and downregulated (20) genes in CPAM compared to normal lung tissues (Supplementary table 1).

**Table 1:** The clinical information of all cases of patients with CPAM.

Case	Gender	Age	Prenatal care	Chest computed tomography (CT) scan before surgery	Respiratory infections
1	Female	6 months 15 days	19 weeks gestation, prenatal ultrasound found a large right-sided solid lung mass, which was considered as CPAM	Multiple cystic lesions in the posterior upper lobe of the right lung, considered as CPAM (type II) (22.4 × 27.0 mm)	0
2	Male	3 months	24 weeks gestation, prenatal ultrasound found a large left-sided solid lung mass, which was considered as CPAM	Multiple cystic lesions in upper lobe of the left lung, considered as CPAM (type I) (37 × 24 × 33 mm)	1
3	Male	4 months 28 days	22 weeks gestation, prenatal ultrasound found a large left-sided solid lung mass, which was considered as pulmonary sequestration	Multiple cystic lesions in lower lobe dorsal and posterior basal segments of the left lung, considered as CPAM (46 × 27 × 42 mm)	1
4	Male	3 months	20 weeks gestation, prenatal ultrasound found a large right-sided solid lung mass, which was considered as CPAM	Multiple cystic lesions in lower lobe of the right lung, considered as CPAM (63 × 52 × 49 mm); poor ventilation of the remaining lungs	2
5	Female	6 months 7 days	28 weeks gestation, prenatal ultrasound found a large left-sided solid lung mass, which was considered as CPAM	Multiple cystic lesions in lower lobe posterior basal segments of the left lung, considered as CPAM (28 × 20 × 22 mm); 2. Localized emphysema in the left lower lobe (20 × 19 × 15 mm)	1
6	Female	5 months 20 days	22 weeks gestation, prenatal ultrasound found a large left-sided solid lung mass, which was considered as pulmonary sequestration	Multiple cystic lesions in lower lobe dorsal and posterior basal segments of the left lung, considered as CPAM (46 × 27 × 42 mm)	1
7	Male	4 months 5 days	20 weeks gestation, prenatal ultrasound found a large right-sided solid lung mass, which was considered as CPAM	Multiple cystic lesions in upper lobe of the right lung, considered as CPAM (40 × 22 × 39 mm)	0
8	Female	7 months 8 days	26 weeks gestation, prenatal ultrasound found a large left-sided solid lung mass, which was considered as CPAM	Multiple cystic lesions in lower lobe posterior basal segments of the left lung, considered as CPAM (38 × 20 × 28 mm)	1
9	Female	6 months 15 days	28 weeks gestation, prenatal ultrasound found a large right-sided solid lung mass, which was considered as CPAM	Multiple cystic lesions in the posterior upper lobe of the right lung, considered as CPAM (type II) (26 × 29 mm)	0
10	Male	4 months 9 days	22 weeks gestation, prenatal ultrasound found a large left-sided solid lung mass, which was considered as CPAM	Multiple cystic lesions in the upper lobe of the left lung, considered as CPAM (type I) (36 × 20 × 35 mm)	1

The gene expression patterns were represented by heat maps (Fig. 2A), and the distinct gene expression profiles in CPAM were displayed by volcano plots (Fig. 2B).

### GO and KEGG function enrichment analyses for the differentially expressed genes

**Figure 3:**
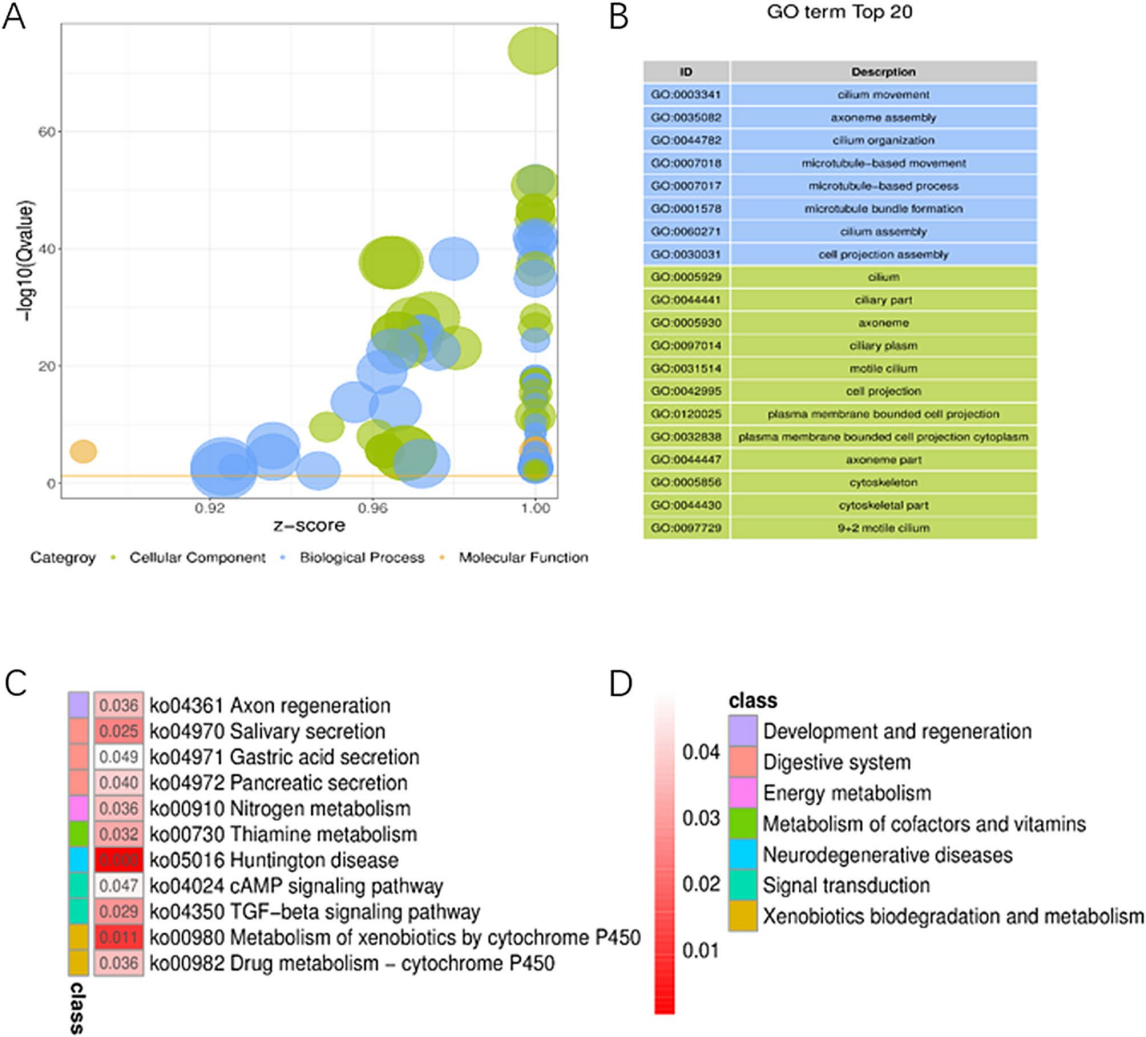
GO and KEGG analysis. (**A**) GO enrichment bubble plot; (**B**) Top 20 GO enrichment terms; (**C**) KEGG pathway enrichment; (**D**) pathway functional classification.

GO enrichment and KEGG pathway analyses were conducted on the 592 DEGs, employing an adjusted *P* value threshold of < 0.05 for screening (Supplementary table 2). In the CC enrichment analysis, these genes exhibited significant enrichment in signaling pathways related to 'cillium' and 'cilliary part' pathways. Regarding BP, the genes were prominently associated with responses to 'cillium movement,' 'axoneme assembly,' and 'cillium organization.' In terms of MF, the genes were primarily involved in binding activities, especially 'heme binding' (Fig. 3A, B. Additionally, KEGG pathway analysis highlighted the 'TGF-β' and 'cAMP' signaling pathways as the most pertinent pathways linked to CPAM (Fig. 3C, D).

### Validation of ciliated epithelium and TGF-β signal pathway related gene in CPAM

**Figure 4:**
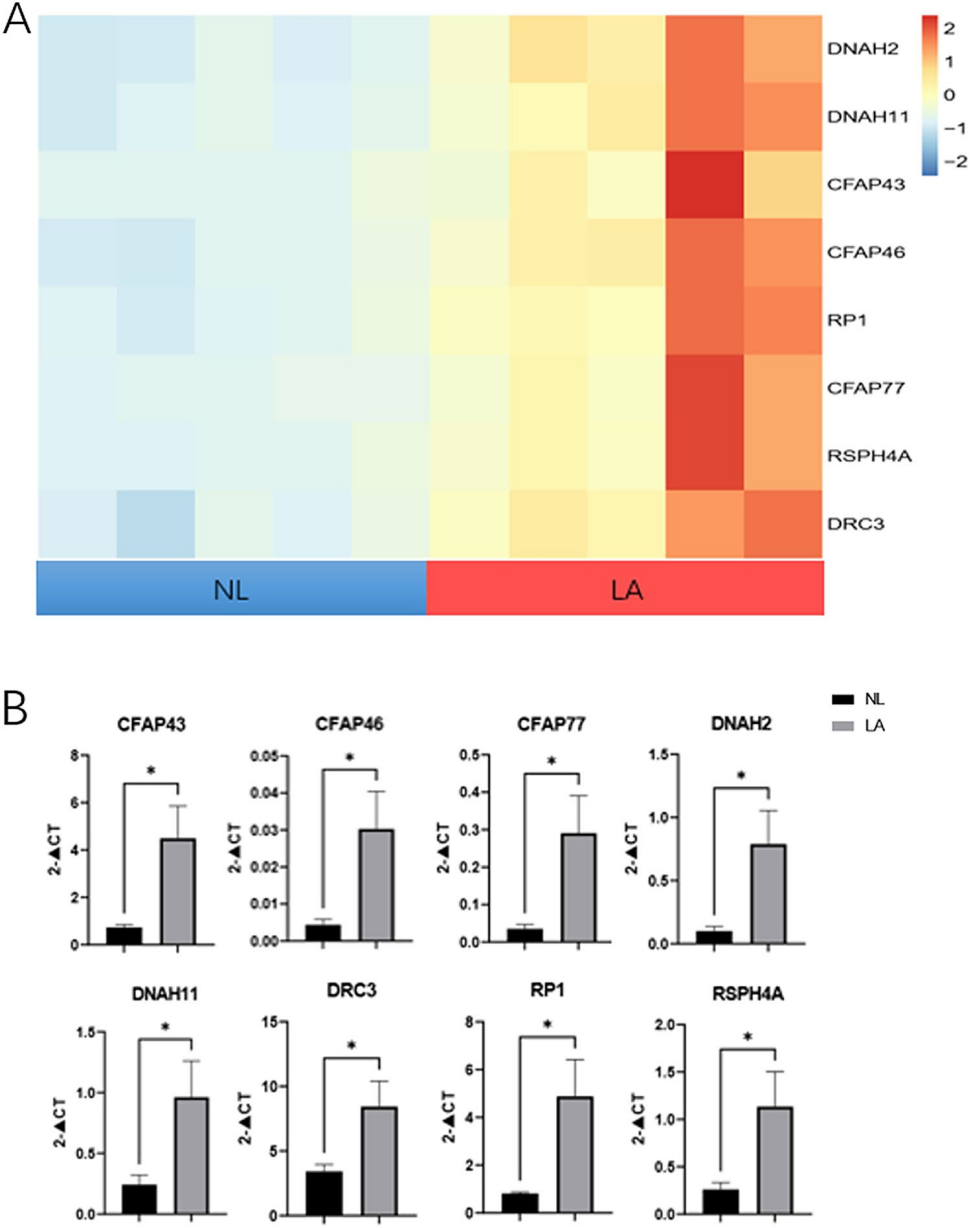
Epithelial gene expression. (**A**) The heatmap displays expression of epithelial-related genes in Non-Lesion (NL) and Lesion (LA) conditions. Rows represent genes, columns represent samples. (**B**) Validation of mRNA expression in tissue samples for epithelial-related genes.

**Figure 5:**
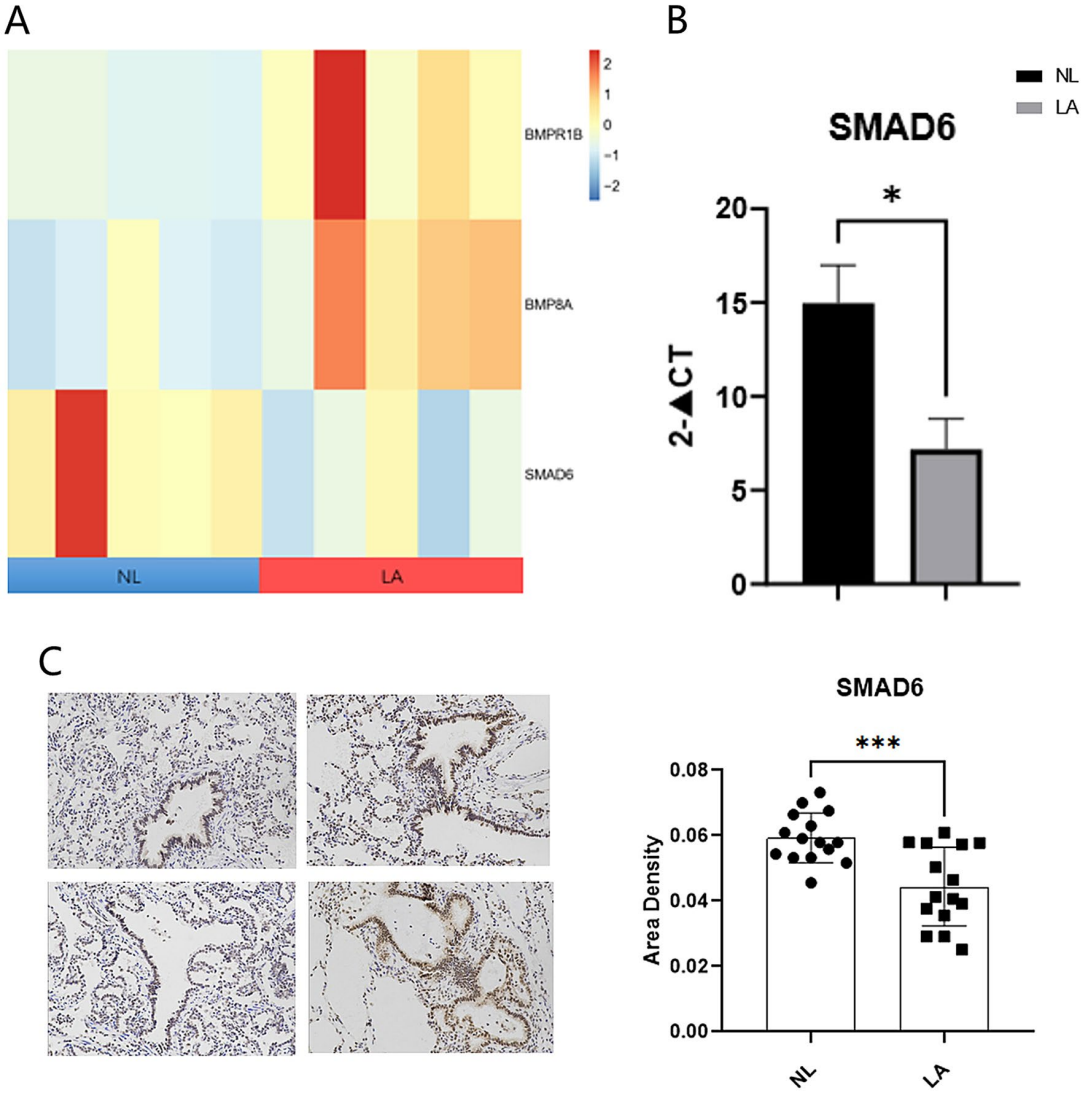
TGF-β signaling pathway differential expression. (**A**) The heatmap displays differential expression of genes in the TGF-β signaling pathway; (**B**) validation of mRNA expression in tissue samples for SMAD6; (**C**) results of the representative immunohistochemistry staining showing the protein level expressions of SMAD6 in NL and LA tissues.

Based on the transcriptome sequencing results, which indicated elevated expression of and dysregulated expression of TGF-β signal pathway in CPAM (Supplementary table 3), further validation experiments were conducted to provide additional support for these findings in tissue samples (Figs. 4A, 5A). We assessed the expression of a panel of genes associated with ciliated epithelium in CPAM tissues. Including DNAH2, DNAH11, CFAP43, CFAP46, RP1, CFAP77, RSPH4A, DRC3, exhibited upregulation in CPAM compared to normal lung tissues (Fig. 4B). These findings suggest these intriguing findings strongly suggest a potential association between the dysregulation of ciliated epithelium-related genes and the pathogenesis of CCAM.

Our investigation also included an examination of genes involved in the TGF-β signaling pathway within CPAM tissues. We observed in the expression of TGF-β signal pathway-related genes, such as SMAD6, when comparing CPAM to adjacent normal lung tissues (Fig. 5B). The downregulation of SMAD6, a critical negative regulator that inhibits the transmission of TGF-β signals, implies the potential for excessive activation of the TGF-β signaling pathway, thesubsequently influencing cell function and tissue development.

### Immunohistochemical analysis of TGF-β signal pathway related genes expression in CPAM

Immunohistochemical analysis was conducted to assess the expression of SMAD6, GDF7, and InhibinB in CPAM tissues, with a particular focus on their potential involvement in the TGF-β signaling pathway. The results revealed a significant decrease in SMAD6 expression, which was a critical negative regulator of the TGF-β signaling pathway. This downregulation of SMAD6 implied a potential for excessive activation of the TGF-β pathway in CPAM (Fig. 5C).

**Figure 6:**
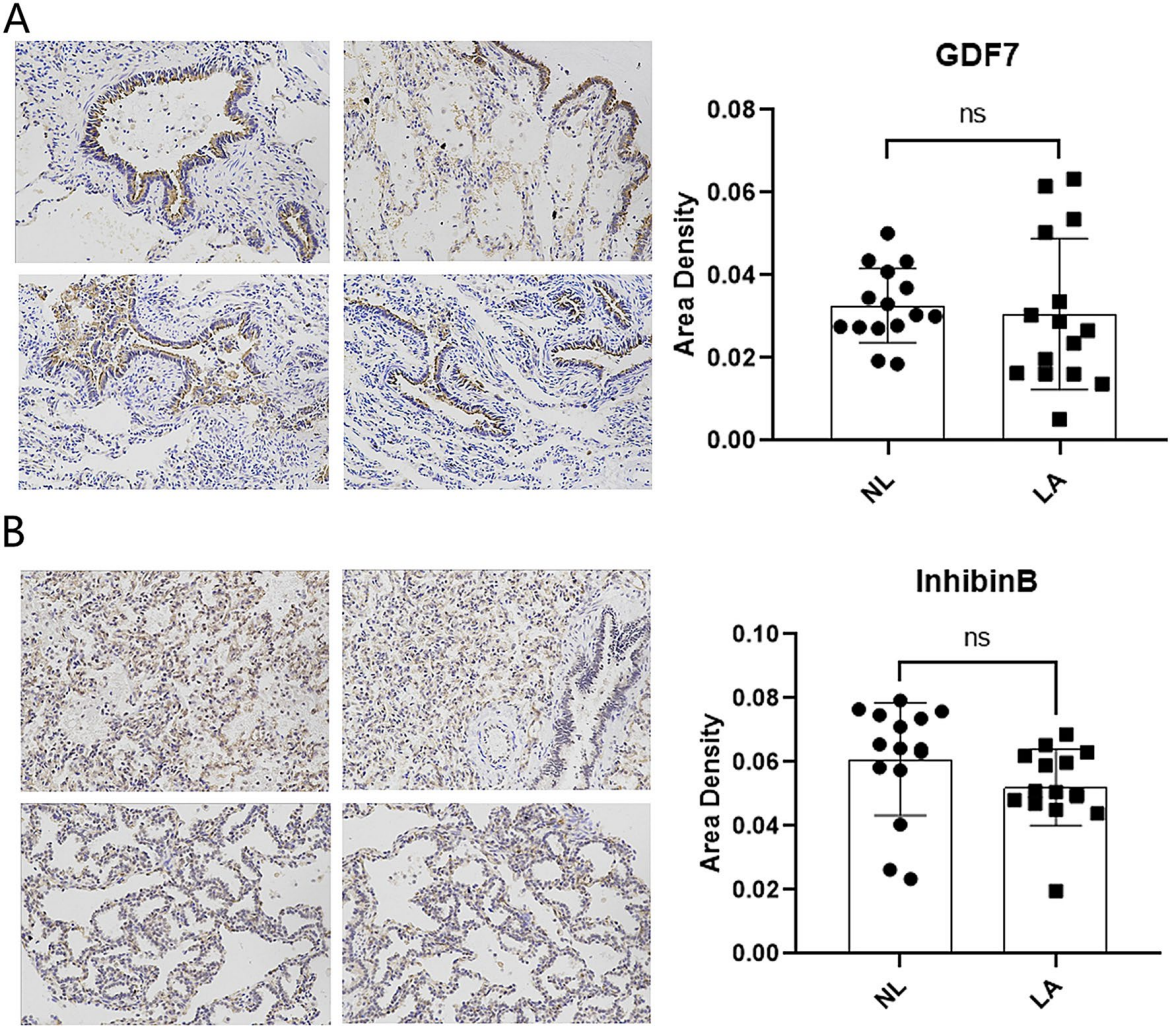
TGF-β signaling pathway related genes expression. (**A**) Results of the representative immunohistochemistry staining showing the protein level expressions of GDF7 in NL and LA tissues; (**B**) results of the representative immunohistochemistry staining showing the protein level expressions of InhibinB in Non-Lesion (NL) and Lesion (LA) tissues.

While GDF7 exhibited a slight increase in expression, but without statistical significance, suggesting a possible association with the TGF-β pathway that warrants further investigation (Fig. 6A). In the case of InhibinB, there was a decreasing trend in expression within CPAM tissues, although statistical significance was not achieved (Fig. 6B). This trend may suggest a role for InhibinB in modulating the TGF-β signaling pathway in CPAM, but additional studies are required to confirm this.

## Discussion

Our study has unveiled a comprehensive overview of the molecular alterations present in Congenital pulmonary airway malformation (CPAM). Through RNA-seq analysis, we identified a substantial number of differentially expressed genes (DEGs) between CPAM and normal lung tissues. These DEGs, comprising both upregulated and downregulated genes, provide a foundation for understanding the underlying genetic landscape of CPAM.

Congenital pulmonary airway malformation has five major subtypes that have been described and assigned by the Stocker classification. Each type originates from a different part of the bronchial tree, subsequently leading to distinct histopathological differentiation, clinical features, malignant potential, and prognosis^1,12^. The overall prognosis for congenital pulmonary airway malformation, when diagnosed prenatally, is excellent. Infection is the most common known complication of congenital pulmonary airway malformation and usually occurs in the first few years of the lives of asymptomatic infants, especially if surgical resection does not take place. The most commonly known malignant complication associated with CPAM is pleuropulmonaryblastoma^13^. Currently, routine prenatal US screening complemented with magnetic resonance imaging (MRI) has become increasingly valuable in detecting CPAMs, with an accuracy of 65–91%^7^. Imaging evaluation and clinical follow-up after birth is required in all cases to confirm the diagnosis and to initiate adequate treatment^14^. Prenatal treatment options include the maternal administration of steroids, minimally invasive procedures or open foetal surgery.

Functional enrichment analyses, including Gene Ontology (GO) and KEGG pathway analyses, have shed light on the potential biological processes and pathways that are dysregulated in CPAM. Notably, the enrichment of ciliated epithelium-related pathways and the TGF-β signaling pathway underscores their potential roles in CPAM pathogenesis.

Furthermore, our validation studies have confirmed the upregulation of ciliated epithelium-associated genes, strengthening the hypothesis that aberrant ciliated epithelium function may contribute to the development of CPAM. Additionally, the downregulation of SMAD6, a critical negative regulator of the TGF-β pathway, suggests the possibility of TGF-β pathway dysregulation in CPAM. The upregulation of genes associated with ciliated epithelium suggests that abnormalities in cilia function may play a role in CPAM development. Dysfunctional cilia can disrupt normal respiratory processes and contribute to lung developmental disorders. Among them, such genes as Dnah2, DNAH11, CFAP43, CFAP77, RSPH4A, and DRC3 play important roles in the driving and regulation of cilia movement^15–20^. The continuous coordinated beating of cilia transports mucus and foreign matter out of the airway to help maintain a sterile environment in the airways, and it takes an essential part in protecting the respiratory tract. Deletion or mutation of one of these genes can lead to alterations in ciliary ultrastructure and ciliary motility, which will lead to poor fiber motility and clearance dysfunction, resulting in severe accumulation of respiratory mucus, eventually leading to inflammation and affecting lung function and development. CFAP46 has also been reported to be associated with airway and lung diseases by modulating inflammatory responses^21^. Moreover, a study showed that the downregulation of ACSL5 and Wnt2B could play an important role in the development of bronchial-alveolar structures in CPAM^22^.

The downregulation of SMAD6, a key negative regulator of the TGF-β signaling pathway, implies excessive activation of this pathway in CPAM^17^. Dysregulated TGF-β signaling can influence cell proliferation, differentiation, and extracellular matrix production, potentially contributing to CPAM pathogenesis^18^. A study showed that TGF-β signaling in prenatal lung mesenchyme is essential for lung development and maturation, and defective TGF-β signaling in lung mesenchyme may be related to abnormal airway branching morphogenesis and congenital airway cystic lesions^23^. Our study lays the foundation for future investigations into CPAM. Detailed functional studies are needed to explore the precise roles of upregulated ciliated epithelium-associated genes in CPAM pathogenesis. Further investigations into the TGF-β signaling pathway in CPAM are essential. This could include assessing downstream signaling events, identifying potential therapeutic targets within the pathway, and elucidating the mechanisms responsible for SMAD6 downregulation. Exploring genetic and epigenetic factors contributing to CPAM susceptibility could provide valuable insights. Our study holds significant implications for the understanding and potential treatment of CPAM. By identifying dysregulated genes and pathways, we offer a stepping stone towards targeted therapies for this congenital lung disorder. Improved clinical management and interventions may arise from unraveling the precise molecular mechanisms driving CPAM.

## Conclusion

In conclusion, this study serves as a crucial initial exploration of the molecular landscape of CPAM, and the downregulation of SMAD6, a critical negative regulator of the TGF-β pathway, suggested the possibility of TGF-β pathway dysregulation in CPAM. In addition, the upregulation of ciliated epithelium-associated genes, strengthening the hypothesis that aberrant ciliated epithelium function may contribute to the development of CPAM.

## Limitation

This study is limited by the relatively small sample size; larger cohorts were considered in the future to enhance the generalizability of the findings. In addition, more validation experiments of Smad-6 in TGF signal pathway in children with CPAM was need.

### Supplementary Information

Supplementary Table 1.

Supplementary Table 2.

Supplementary Table 3.

## Data Availability

The data could be shared after publication upon request for use via communication with the corresponding authors.

## Abbreviations

CPAM: Congenital pulmonary malformation
cDNA: Complementary DNA
DEGs: Differentially expressed genes
FGF-10: Fibroblast growth factor-10
GO: Gene ontology
NGS: Next Generation Sequencing
NL: Normal lung tissue
qRT-PCR: Quantitative reverse transcriptase polymerase chain reaction
RIN: RNA integrity number
rRNA: Ribosomal RNA
TGF-β: Transforming growth factor-β
